# Protective role for miR-9-5p in the fibrogenic transformation of human dermal fibroblasts

**DOI:** 10.1186/s13069-016-0044-2

**Published:** 2016-05-10

**Authors:** Verónica Miguel, Oscar Busnadiego, Marta Fierro-Fernández, Santiago Lamas

**Affiliations:** Department of Cell Biology and Immunology, Centro de Biología Molecular “Severo Ochoa”, Consejo Superior de Investigaciones Científicas–Universidad Autónoma de Madrid, Nicolás Cabrera 1, 28049 Madrid, Spain

**Keywords:** Skin fibrosis, miR-9-5p, Myofibroblasts, TGF-β signaling

## Abstract

**Background:**

Excessive accumulation of extracellular matrix (ECM) proteins is the hallmark of fibrotic diseases, including skin fibrosis. This response relies on the activation of dermal fibroblasts that evolve into a pro-fibrogenic phenotype. One of the major players in this process is the cytokine transforming growth factor-β (TGF-β). MicroRNAs (miRNAs) are small non-coding RNAs that post-transcriptionally regulate gene expression affecting a wide range of pathophysiological events including fibrogenesis. MicroRNA-9-5p (miR-9-5p) has been shown to exert a protective role in lung and peritoneal fibrosis. This study aimed to evaluate the role of miR-9-5p in skin fibrosis.

**Results:**

miR-9-5p is up-regulated in TGF-β1-treated human dermal fibroblasts (HDFs). In silico identification of miR-9-5p targets spotted the type II TGF-β receptor (TGFBR2) as a potential TGF-β signaling-related effector for this miRNA. Consistently, over-expression of miR-9-5p in HDFs down-regulated TGFBR2 at both the mRNA and protein levels and reduced the phosphorylation of Smad2 and the translocation of Smad2/3 to the nucleus. In keeping, over-expression of miR-9-5p significantly delayed TGF-β1-dependent transformation of dermal fibroblasts, decreasing the expression of ECM protein collagen, type I, alpha 1 (Col1α1), and fibronectin (FN), the amount of secreted collagen proteins, and the expression of the archetypal myofibroblast marker alpha-smooth muscle actin (α-SMA). By contrast, specific inhibition of miR-9-5p resulted in enhanced presence of fibrosis markers. The expression of miR-9-5p was also detected in the skin and plasma in the mouse model of bleomycin-induced dermal fibrosis. Using lentiviral constructs, we demonstrated that miR-9-5p over-expression was also capable of deterring fibrogenesis in this same model.

**Conclusions:**

miR-9-5p significantly prevents fibrogenesis in skin fibrosis. This is mediated by an abrogation of TGF-β-mediated signaling through the down-regulation of TGFBR2 expression in HDFs. These results may pave the way for future diagnostic or therapeutic developments for skin fibrosis based on miR-9-5p.

**Electronic supplementary material:**

The online version of this article (doi:10.1186/s13069-016-0044-2) contains supplementary material, which is available to authorized users.

## Background

Fibrosis is a process characterized by the excessive synthesis and deposition of extracellular matrix (ECM) proteins. It can affect several organs leading to the progressive replacement of functional tissue. In the skin, fibrosis develops in the dermis, the connective tissue layer under the basement membrane and the epidermis and is mainly composed by ECM. The latter is made of fibrous structural proteins such as collagen and elastin, adhesive glycoproteins (fibronectin (FN) and laminin), proteoglycans, and hyaluronan. Together, they provide structural and biomechanical support to cellular components, mostly fibroblasts. ECM remodeling is mediated by lysyl oxidases (LOXs) that cross-link collagens and elastins through oxidative deamination of lysine residues, whereas ECM dynamic degradation is mainly mediated by the endopeptidase matrix metalloproteases (MMPs) [1]. Skin fibrosis is a hallmark feature of several diseases, including systemic sclerosis (SSc), keloids, hypertrophic scars, and graft-versus-host disease [2]. To date, no therapy has been shown to reverse or arrest the progression of skin fibrosis, even though it has a profound impact on the duration and quality of life. For instance, systemic sclerosis affects 100,000 people in the USA with a median survival time of 11 years [3]. Despite their different etiological origin, these diseases converge in a common aberrant wound repair response [4]. Transforming growth factor-β (TGF-β) and other pro-fibrotic cytokines, which are released in this persistent inflammation response, lead to the activation, proliferation, and differentiation of fibroblasts into myofibroblasts, the cell type ultimately responsible for the synthesis, remodeling, and contraction of ECM [5]. Myofibroblasts are commonly identified by the expression of alpha-smooth muscle actin (α-SMA), and they are characterized by a pronounced rough endoplasmic reticulum, stress fibers, and a large nucleus. Although they mainly derive from resident fibroblasts, they can be recruited from skin-derived precursors and bone marrow fibrocytes. In some tissues, epithelial and endothelial to mesenchymal transition (EMT and EndoMT, respectively), vascular smooth muscle cells, and pericytes may also contribute to fibroblast accumulation [6].

One of the major mediators in the fibrogenic processes, TGF-β, has been also implicated in the pathogenesis of skin fibrotic disorders [7]. SSc patients show high levels of plasma TGF-β, and fibroblasts from these patients display elevated levels of TGF-β expression [8, 9]. In several established animal models of SSc, TGF-β acts as a key mediator in fibrosis development and the reduction of TGF-β expression by several therapeutic strategies shows anti-fibrotic effects [10–13]. TGF-β acts by interacting with a heteromeric complex of transmembrane serine/threonine kinase receptors, the type I TGF-β receptor (TGFBR1) and type II TGF-β receptor (TGFBR2). The activation of the type I receptor leads to the propagation of signaling by at least two independent routes: the Smad-dependent canonical pathway and the Smad-independent or non-canonical pathways. In the first one, the activation of TGFBR1 leads to phosphorylation of receptor-specific Smad (R-Smad) proteins, Smad2 and Smad3. Upon phosphorylation, R-Smads together with the common-mediator Smad (co-Smad), Smad4, translocate to the nucleus, where they interact with other transcription factors (co-factors) to regulate transcriptional responses [14]. Consequently, dysregulated TGF-β signaling has been reported in skin fibrotic diseases. It was reported that fibroblasts from SSc patients show an increase of TGF-β receptors and Smad2/3 nuclear translocation, while Smad7 expression is reduced [15]. In keloid fibroblasts, increased expression of TGFBR1 and TGFBR2 and increased phosphorylation of Smad3 are present, supporting a central role for TGF-β/Smad signaling in its pathogenesis [16].

MicroRNAs (miRNAs) are a group of small (~19–24 nucleotides), non-coding RNAs that modulate gene expression by interacting with the 3′-untranslated region (3′-UTR) of the corresponding target gene messenger RNA (mRNA) [17, 18]. In most circumstances, miRNAs are believed to either repress mRNA translation or reduce mRNA stability [17, 18]. miRNAs are proposed to promote cellular robustness and homeostasis, and hence, alterations of miRNA expression can occur as a response to stress-related phenomena in a variety of diseases [19]. These small RNAs have been described to play a major role in the initiation and progression of numerous diseases, including the fibrosis of several organ systems, such as the skin, heart, liver, kidney, and lungs [20, 21]. In the context of skin fibrosis, it is well illustrated that miRNAs are involved in this disease by regulating TGF-β signaling, ECM synthesis, and degradation, by controlling the proliferation and differentiation of myofibroblasts. They have been also implicated in the EMT process as well as in the pathogenesis and maintenance of skin fibrosis [3, 22]. Among these, miR-29 has been extensively studied and is the best characterized negative regulator of ECM protein synthesis by directly targeting collagen, type I, alpha 1 (Col1α1) mRNA [23–26]. SSc patient-derived skin fibroblasts showed a consistent down-regulation of miR-29 [27], and over-expression of this miRNA has been proposed as a potent anti-fibrotic strategy [23, 25]. In addition to miR-29, several other miRNAs are known to negatively regulate collagen expression, such as let-7a and miRNA-196a, both down-regulated in SSc [28, 29]. The expression of miR-21 was increased in both SSc skin tissues and fibroblasts [26]. This miRNA is up-regulated by TGF-β signaling, and miR-21 in turn reduces the expression of Smad7, releasing the inhibition of the TGF-β pathway, thus creating a feed-forward loop [30]. Let-7g and miR-23b up-regulate TGFBR2 expression and are associated with SSc [31], while miR-140-5p, miR-17-5p, and miR-20 have an opposite effect, thus resulting in hypertrophic scars [32]. In the clinical setting, miRNAs may be considered potential biomarkers due to their stability and relatively easy detection in biological fluids. Importantly, due to the poor efficacy of available treatments like cyclophosphamide, prednisolone, or methotrexate against skin fibrosis, the development of new therapeutic approaches is urgent. miRNA-targeted therapies attempt to increase the expression of anti-fibrotic miRNAs by miRNA mimics or viral vectors with built-in miRNA precursors or to reduce the expression of pro-fibrotic miRNAs using anti-miRNAs, sponges, erasers, and masks [33].

We have recently reported the protective role of microRNA-9-5p (miR-9-5p) in lung and peritoneal fibrosis [34]. Human miR-9 is encoded by three distinct genomic loci, located in chromosomes 1, 5, and 15, generating three mature miR-9 species with identical sequences. This miRNA is broadly conserved in animals and was first identified as a crucial regulator for the development and maturation of the nervous system in vertebrates [35] and in human brain pathologies [36, 37] as well as in tumors outside the nervous system [38–40]. Noteworthy, there is no information, to our knowledge, implicating miR-9-5p in skin fibrosis. This study aimed to analyze the potential role of miR-9-5p in skin fibrosis by using human dermal fibroblasts (HDFs) in culture and a mouse model of bleomycin-induced skin fibrosis. We found that miR-9-5p prevents the transformation of human fibroblasts into myofibroblasts by blocking TGF-β signaling and significantly attenuates bleomycin-induced skin fibrosis.

## Results

### miR-9-5p is induced by TGF-β1 and reduces TGFBR2 expression in human dermal fibroblasts

**Fig. 1:**
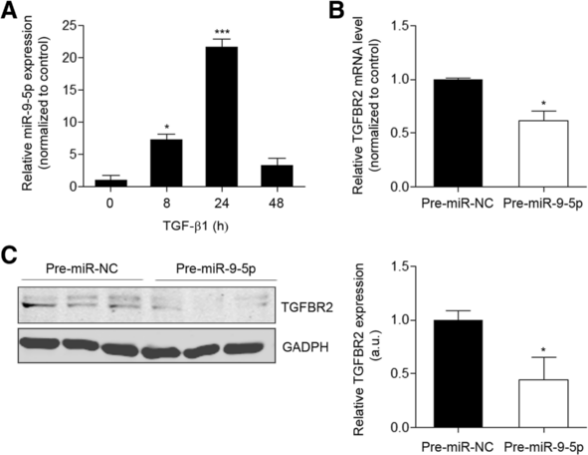
miR-9-5p is induced by TGF-β1 and reduces TGFBR2 expression. **a** miR-9-5p expression was assayed by qRT-PCR in HDFs stimulated with TGF-β1 (5 ng/ml) at the indicated times. **b** qRT-PCR analysis for mRNA expression of TGFBR2 in HDFs transfected with 40-nM pre-miR-9-5p or pre-miR-NC. **c** Western blot analysis (*left*) and quantification (*right*) of TGFBR2 expression in HDFs transfected with 40-nM pre-miR-9-5p or pre-miR-NC (a.u., arbitrary units). The *bar graph* shows values after correction by GAPDH expression and normalized to control conditions. The *bar graphs* show mean ± SEM of three independent experiments, *P < 0.05 and ***P < 0.001 compared to control cells

To study the possible implication of miR-9-5p in skin fibrosis, HDFs were treated with 5 ng/ml TGF-β1 for the indicated times and its expression was analyzed by quantitative reverse transcription-polymerase chain reaction (qRT-PCR). TGF-β1 induced a time-dependent over-expression of this miRNA, with an increase of more than 20 times after 24-hour (h) treatment compared to control cells (Fig. 1a). This result suggests the potential involvement of miR-9-5p in the TGF-β1-related signaling responses in dermal fibroblasts. TGFBR2 has been previously validated as a target of miR-9-5p [34]. In silico analysis of the 3′-UTR shows that it bears two seed target sites for miR-9-5p, one poorly and the other well conserved across several vertebrate species. To validate if miR-9-5p also modulates TGFBR2 expression in HDFs and to determine if this miRNA affects its mRNA stability and/or its translation, we performed gain-of-function experiments. Over-expression of miR-9-5p in dermal fibroblasts resulted in a significant decrease of about 50 % in both TGFBR2 mRNA and protein levels (Fig. 1b, c), indicating the negative regulation of TGFBR2 by miR-9-5p in HDFs. TGFBR1 is also an in silico predicted target of miR-9-5p. However, the over-expression of this miRNA did not alter TGFBR1 mRNA levels (Additional file 1: Figure S1A).

### miR-9-5p attenuates TGF-β1 pro-fibrogenic signaling in human dermal fibroblasts

**Fig. 2:**
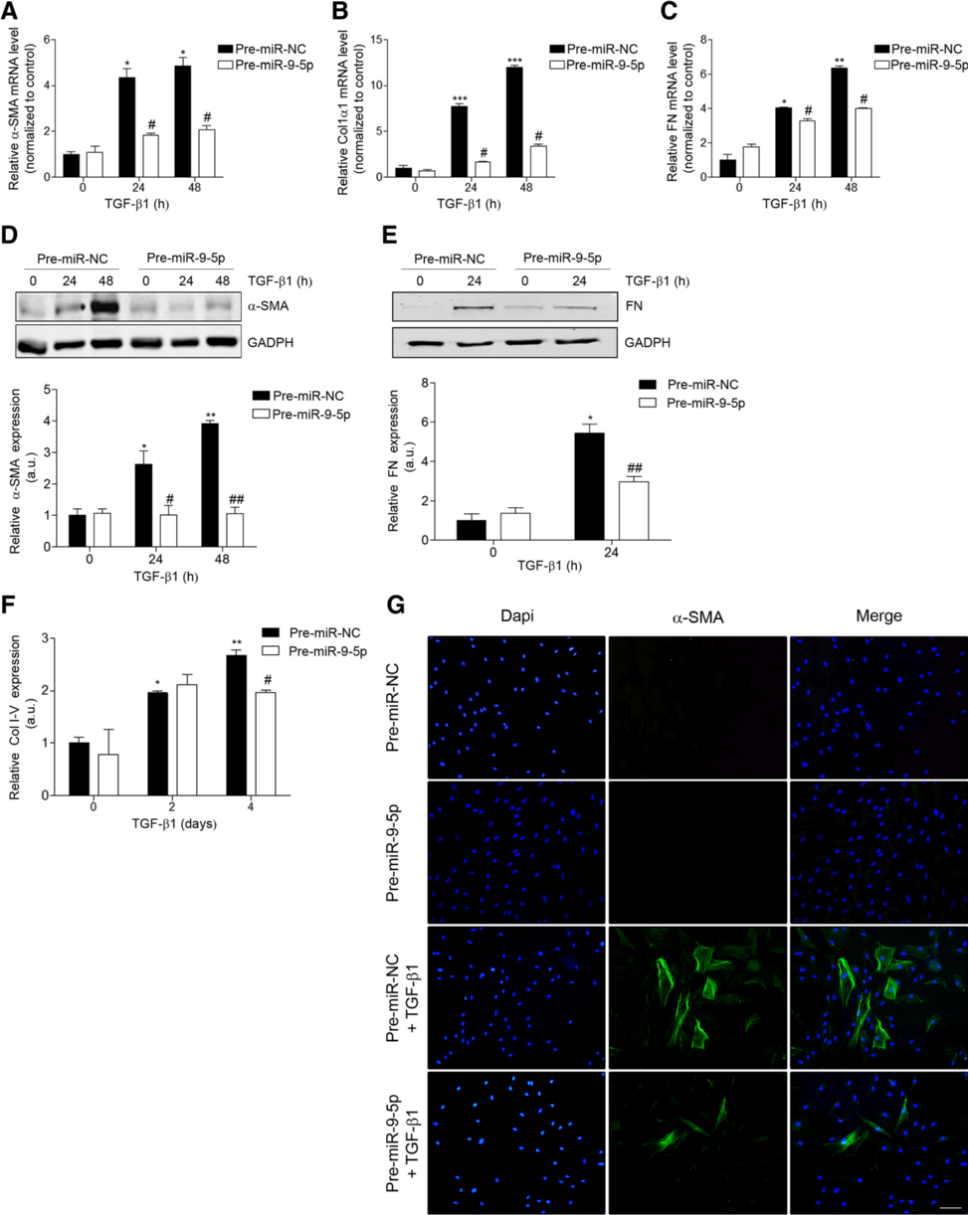
miR-9-5p over-expression abrogates TGF-β1-induced differentiation of human dermal fibroblasts into myofibroblasts. **a**–**c** qRT-PCR analysis for mRNA expression of α-SMA (**a**), Col1α1 (**b**), and FN (**c**) in HDFs transfected with 40-nM pre-miR-9-5p or pre-miR-NC and treated with TGF-β1 (5 ng/ml) for the indicated times. **d** Western blot analysis (*top*) and quantification (*bottom*) of α-SMA levels in HDFs transfected with 40-nM pre-miR-9-5p or pre-miR-NC and treated with TGF-β1 (5 ng/ml) for the indicated times (a.u., arbitrary units). The *bar graph* shows values after correction by GAPDH expression and normalized to control conditions. **e** Western blot analysis (*top*) and quantification (*bottom*) of FN levels in HDFs transfected with 40-nM pre-miR-9-5p or pre-miR-NC and treated with TGF-β1 (5 ng/ml) for the indicated times (a.u., arbitrary units). The *bar graph* shows values after correction by GAPDH expression and normalized to control conditions. **f** Sircol assay of secreted collagen proteins I-V was done in HDFs transfected with 40-nM pre-miR-9-5p or pre-miR-NC and treated with TGF-β1 (5 ng/ml) for the indicated times (a.u., arbitrary units). **g** Immunofluorescence staining of α-SMA (*green*) in HDFs treated with TGF-β1 (5 ng/ml) for 48 h after transfection with 40-nM pre-miR-9-5p or pre-miR-NC. Nuclei were stained with DAPI (*blue*). *Scale bars*: 100 μm. The *bar graphs* show mean ± SEM of three independent experiments, *P < 0.05, **P < 0.01, and ***P < 0.001 compared to control cells and ^#^ *P* < 0.05 and ^##^ *P* < 0.01 compared to its corresponding negative control time point

**Fig. 3:**
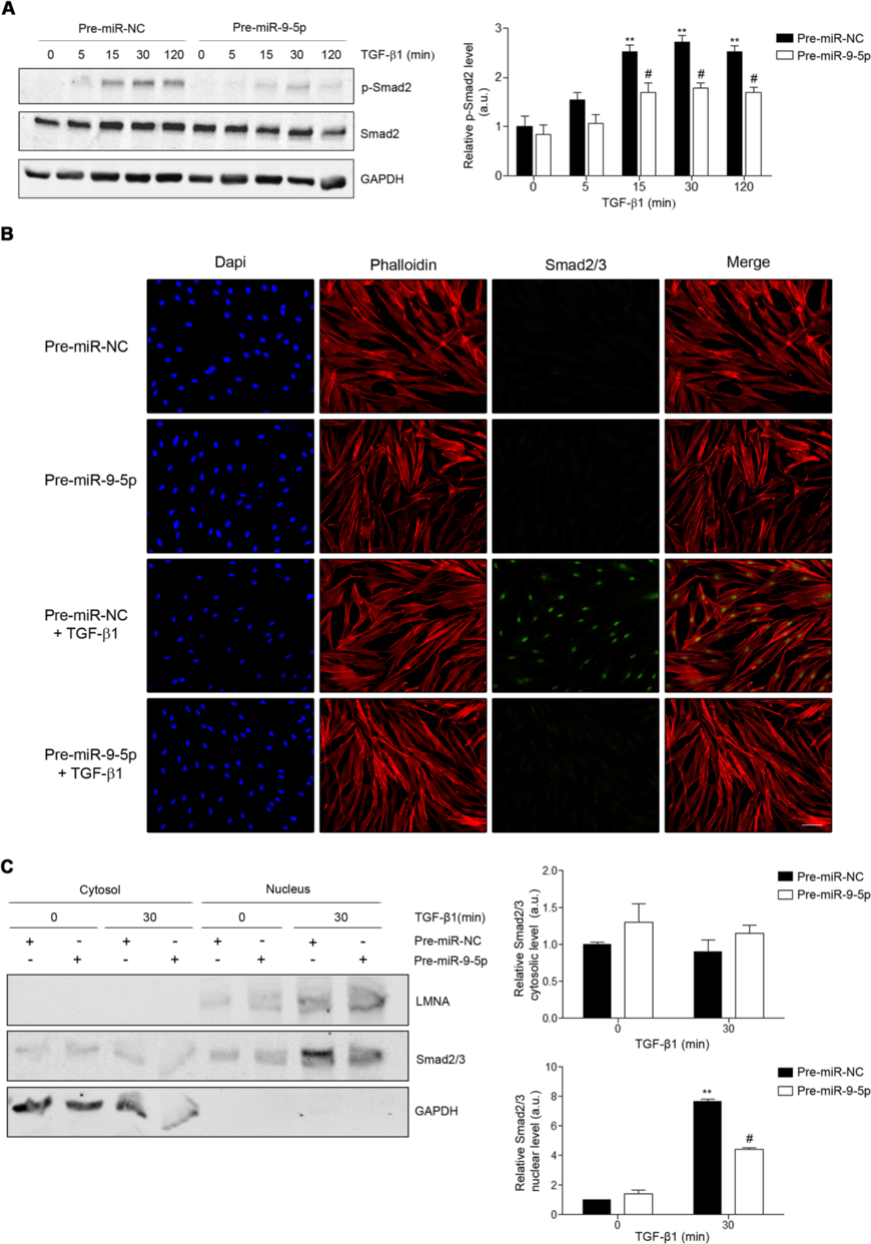
miR-9-5p attenuates Smad-dependent TGF-β signaling. **a** Western blot analysis (*left*) and quantification (*right*) of pSmad2 protein levels in HDF cells transfected with 40-nM pre-miR-9-5p or pre-miR-NC and treated with TGF-β1 (5 ng/ml) at the indicated times (a.u., arbitrary units). The *bar graph* shows values after correction by Smad2 expression and normalized to control conditions. **b** Immunofluorescence staining of Smad2/3 (*green*) in HDFs treated with TGF-β1 (5 ng/ml) for 30 min after transfection with 40-nM pre-miR-9-5p or pre-miR-NC. Nuclei were stained with DAPI (*blue*), and F-actin was stained with phalloidin (*red*). *Scale bars*: 100 μm. **c** Western blot analysis after nuclear/cytoplasmic fractionation (*left*) and quantification (*right*) of Smad2/3 protein levels in HDF cells transfected with 40-nM pre-miR-9-5p or pre-miR-NC and treated with TGF-β1 (5 ng/ml) at the indicated times (a.u., arbitrary units). The *bar graph* shows values after correction by GAPDH (cytosol) and LMNA (nucleus) expression and normalized to control conditions. All *bar graphs* represent mean ± SEM of three independent experiments, **P < 0.01 compared to control cells and ^#^ *P* < 0.05 compared to its corresponding negative control time point

**Fig. 4:**
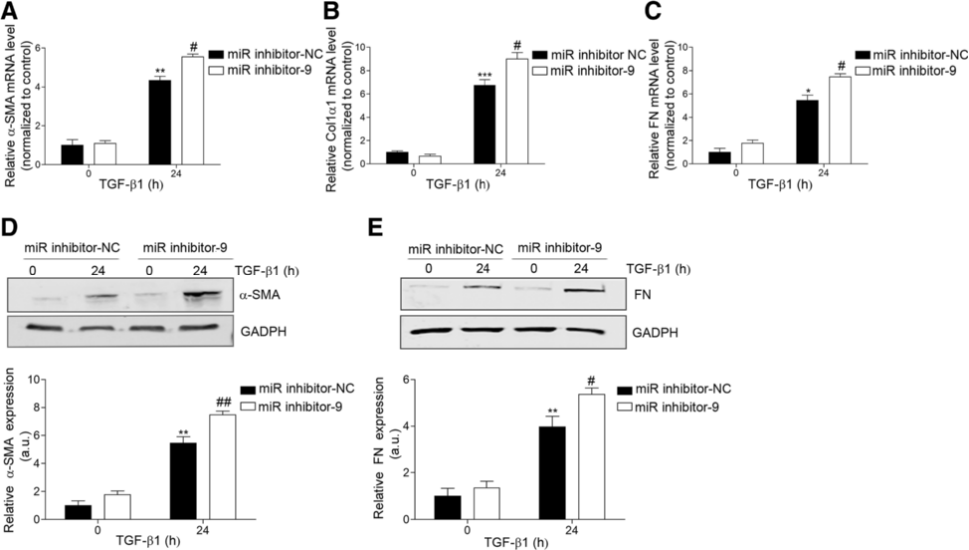
Inhibition of miR-9-5p enhances TGF-β1-induced transformation of human dermal fibroblasts into myofibroblasts. **a**–**c** qRT-PCR analysis for mRNA expression of α-SMA (**a**), Col1α1 (**b**), and FN (**c**) in HDF transfected with 40-nM miR inhibitor-NC or miR inhibitor-9 and treated with TGF-β1 (5 ng/ml) for the indicated times. **d** Western blot analysis (*left*) and quantification (*right*) of α-SMA and FN levels in HDF transfected with 40-nM miR inhibitor-9 or miR inhibitor-NC (control) and treated with TGF-β1 (5 ng/ml) for the indicated times (a.u., arbitrary units). The *bar graphs* show values after correction by GAPDH expression and normalized to control conditions. **e** Western blot analysis (*top*) and quantification (*bottom*) of FN levels in HDF transfected with 40-nM miR inhibitor-9 or miR inhibitor-NC (control) and treated with TGF-β1 (5 ng/ml) for the indicated times (a.u., arbitrary units). The *bar graph* shows values after correction by GAPDH expression and normalized to control conditions. The *bar graphs* show mean ± SEM of three independent experiments, *P < 0.05, **P < 0.01, and ***P < 0.001 compared to control cells and ^#^ *P* < 0.05 and ^##^ *P* < 0.01 compared to their corresponding negative control time point

To assess if miR-9-5p-related reduction in TGFBR2 expression was associated with a decrease in the TGF-β-induced pro-fibrogenic transformation of fibroblasts, HDFs were transfected with 40-nM precursor of miR-9-5p (pre-miR-9-5p) and incubated with 5 ng/ml TGF-β1 for different times. As shown in Fig. 2, over-expression of miR-9-5p significantly abolished TGF-β1-induced transcription of α-SMA, Col1α1, and FN after 24- and 48-h treatments (Fig. 2a–c). Noticeably, the abrogation was almost complete in the case of Col1α1 (Fig. 2b). Similarly, increasing levels of miR-9-5p strongly reduced TGF-β1-induced α-SMA, FN, and collagen type I-V protein abundance (Fig. 2d–f), as well as the amount of α-SMA-positive fibroblasts in the presence of TGF-β1 for 24 h (Fig. 2g). These data suggest that miR-9-5p can prevent the TGF-β1-dependent differentiation of dermal fibroblasts into myofibroblasts by regulating the TGF-β signaling pathway. Consistently, miR-9-5p significantly abrogated the phosphorylation of Smad2 and the translocation of Smad2/3 to the nucleus that usually occurs after TGF-β1 stimulation, visible after both short and prolonged periods of exposure (Fig. 3a–c, Additional file 1: Figure S1C), without affecting the expression of the inhibitory Smad, Smad7 (Additional file 1: Figure S1B). Loss-of-function experiments with the miR inhibitor-9 showed an up-regulation of the TGF-β1-induced transcription of α-SMA, Col1α1, and FN after 24-h treatment (Fig. 4a–c). This increase was also observed on α-SMA and FN protein levels (Fig. 4d, e). Altogether, these results suggest that miR-9-5p exerts an inhibitory effect on TGF-β1-dependent fibroblast differentiation, which is mediated, at least in part, by the negative regulation of the Smad-dependent pathway related to TGF-β signaling.

### miR-9-5p prevents bleomycin-induced skin fibrosis

**Fig. 5:**
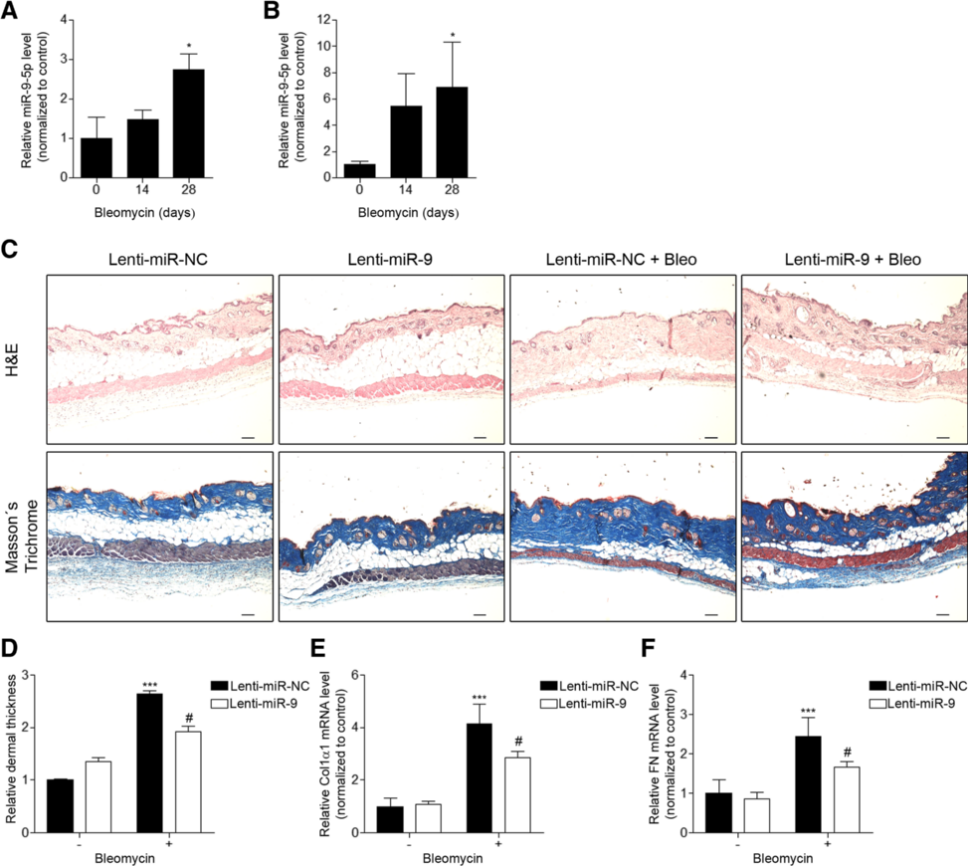
miR-9-5p prevents bleomycin-induced skin fibrosis. **a**, **b** miR-9-5p expression was assayed by qRT-PCR in the skin (**a**) and plasma (**b**) after daily subcutaneous injections of 10 μg bleomycin for 14 and 28 days. **c** H&E staining and Masson’s trichrome blue staining for collagen in skin samples from mice administered 1 × 10^8^ ifu of lenti-SC (control) or lenti-miR-9 for 4 days followed by daily subcutaneous injections of 10 μg bleomycin for 4 weeks. *Scale bars*: 100 μm. **d** Quantitative evaluation of dermal thickness from the same samples as in (**c**), calculated as the ratio between dermis and total skin thickness. **e**, **f** qRT-PCR analysis for mRNA expression of Col1α1 (**e**) and FN (**f**) in skin samples from mice administered 1 × 10^8^ ifu of lenti-SC (control) or lenti-miR-9 for 4 days followed by daily subcutaneous injections of 10 μg bleomycin for 4 weeks. All *bar graphs* represent mean ± SEM (*n* = 6 mice in each group), *P < 0.05 and ***P < 0.001 compared to mice given control lentivirus and saline-treated and ^#^ *P* < 0.05 compared to mice given control lentivirus and bleomycin-treated

The above results prompted us to investigate whether the increased levels of miR-9-5p in HDFs after TGF-β treatment were recapitulated in the SSc murine model of bleomycin-induced skin fibrosis. To determine the expression level of miR-9-5p in skin samples of bleomycin-treated mice, bleomycin was injected subcutaneously and its effect analyzed after different time periods. Analysis by qRT-PCR showed that miR-9-5p was significantly up-regulated approximately threefold in skin samples from animals after 28 days of bleomycin treatment compared to those from untreated animals (Fig. 5a), indicating the possible implication of this miRNA in SSc. Recent studies indicate that miRNAs in body fluids can represent novel biomarkers for various diseases [41]. These circulating miRNAs are found to be remarkably stable even under harsh conditions of temperature or pH [42]. Interestingly, circulating miRNAs are protected from endogenous RNase activity [43], possibly due to packaging in microparticles or apoptotic bodies, to association with RNA-binding proteins or by linkage to high-density lipoproteins [44]. Furthermore, secreted miRNAs can be delivered into recipient cells where they function as endogenous miRNAs, simultaneously regulating multiple target genes or signaling pathways [44, 45]. Thus, serum miRNA levels may not merely be secreted from apoptotic cells but may exert some biological effects in cell-to-cell communication. We evaluated the possibility that plasma miR-9-5p levels can be a disease marker in the mouse model of SSc. An increase of miR-9-5p expression of approximately sixfold was observed in plasma samples from mice after 28 days of bleomycin treatment compared to those from untreated animals (Fig. 5b), suggesting that this miRNA could be a novel biomarker for SSc. To determine the possible effect of this miRNA in the development of experimental dermal fibrosis, lentiviral vectors expressing a scramble negative control construct (lenti-SC) or miR-9-5p (lenti-miR-9) were subcutaneously administrated in mice 4 days before bleomycin administration. To analyze the extent of skin fibrosis induced by bleomycin, histological analysis was performed. An increase in dermal thickness and accumulation of collagen replacing the subcutaneous adipose layer were observed in mice injected subcutaneously with bleomycin for 4 weeks compared to control mice, as examined by hematoxylin and eosin (H&E) and Masson’s trichrome staining, respectively (Fig. 5c). Importantly, pre-administration of lentiviral vectors carrying miR-9-5p effectively attenuated dermal thickness (Fig. 5d). Consequently, lenti-miR-9 administration also significantly reduced the elevated expression of ECM-related genes, such as Col1α1 and FN, elicited by bleomycin administration (Fig. 5e, f). All these results together suggest a possible protective role for miR-9-5p in skin fibrosis and a possible use of this miRNA as a diagnostic marker for this condition.

## Discussion

Given our previously reported observations on the anti-fibrotic action of miR-9-5p in lung and peritoneal fibrosis, the goal of this study was to determine whether miR-9-5p might play a similar role in the prevention of skin fibrosis and fibroblast fibrogenic transformation. We found that miR-9-5p expression was up-regulated 20-fold in HDFs after their treatment with TGF-β1. In keeping, miR-9-5p has been reported to be up-regulated in dermal fibroblasts isolated from SSc patients [28] and in TGF-β-treated lung fibroblasts and omentum-derived mesenchymal cells [34]. This regulation of miR-9-5p by TGF-β could potentially occur via primary miRNAs (pri-miRNAs) due to the in silico identification of at least two Smad-binding elements (SBEs) in the putative promoter region of miR-9-1 and at least another two for miR-9-3 [34]. However, other regulatory mechanisms cannot be excluded. TGF-β is not only able to regulate gene expression at the level of transcription but also to control Drosha-mediated miRNA processing [46]. In addition, it is possible that other specific transcription factors like REST, which mediate the expression of this miRNA in other cell types, could be regulated by TGF-β and may participate in this response [47]. TGFBR2 was previously proposed by our group as a direct target of miR-9-5p not only by in silico analysis and further molecular validation but also by the reduced action of this miRNA in the presence of exogenously enhanced expression of this protein [34]. TGFBR2 is a transmembrane serine/threonine kinase receptor necessary for TGF-β signal transduction whose 3′-UTR contains two target sites for miR-9-5p, of which one is highly conserved among vertebrates and has been implicated in the development of fibrotic processes. In the skin, TGFBR2 expression is increased in SSc fibroblasts as well as in keloids [15, 16]. Consistently, miR-9-5p down-regulated TGFBR2 expression at both the mRNA and protein levels by approximately 50 % in HDFs. This suggests that both mRNA translation repression and degradation mechanisms could be involved in the negative regulation of the expression of TGFBR2 by miR-9-5p. TGFBR2 down-regulation constitutes one of the plausible mechanisms to explain the anti-fibrotic action of miR-9-5p. However, it is possible that other targets related with TGF-β signaling pathway and/or ECM synthesis can also contribute to this outcome. Examination of miR-9-5p function revealed that over-expression of this miRNA followed by treatment with TGF-β1 decreased the abundance of α-SMA-positive fibroblasts and the α-SMA and ECM-related gene, Col1α1 and FN, expression. The higher degree of inhibition of Col1α1 transcription compared to that of FN could be attributed to the fact that Col1α1 is a potential direct target of miR-9-5p according to TargetScan database [48]. Moreover, increasing levels of miR-9-5p also abrogated Smad2 phosphorylation and nuclear translocation of Smad2/3. Additionally, miR inhibitor-9 increased TGF-β1-induced expression of α-SMA, Col1α1, and FN. Consistently, Smad-dependent genes such as Col1α1 resulted more affected in vivo and in vitro than those regulated by Smad-independent pathways, including FN [49–51]. As an individual miRNA may modulate multiple target genes, the potential effect of miR-9-5p on other targets aside from TGFBR2 down-regulation cannot be excluded as part of the anti-fibrotic response. Although we excluded the effect of miR-9-5p on TGFBR1, other potential in silico TGF-β-related targets such as transforming growth factor, beta-induced (TGFBI), Smad4, or NADPH oxidase 4 (NOX4) remain as potential unexplored proteins whose functional inhibition could account for part of the observed effects. Of interest, NOX4 is involved in the generation of reactive oxygen species (ROS) that contribute to the activation of latent TGF-β [52]. Moreover, it is conceivable that the inhibition of this signaling pathway could have several side effects on other biological processes modulated by TGF-β. Additionally, other miR-9-5p-predicted target genes implicated in alternative signaling activated during skin fibrosis, such as the Wnt/β-catenin pathway, or related to the inflammatory immune response may also form part of the mechanisms underlying miR-9-5p action. In this regard, reported targets of miR-9 in other cell lines such as E-cadherin or the transcription factor SOX2 have been shown to regulate the Wnt pathway [53, 54]. Similarly, MMP-13 and MMP-14 are validated miR-9-5p targets that are involved in ECM degradation [55, 56]. In consistence with *in cellulo* results and beyond the caveats in the mouse model to reproduce some skin fibrotic diseases [57], by using lentiviral vectors containing miR-9-5p precursors, we found significant abrogation of dermal fibrogenesis. Histological and expression analysis revealed that in vivo miR-9-5p over-expression promoted attenuation of the bleomycin-induced increase in dermal thickness measured by accumulation of collagen.

**Fig. 6:**
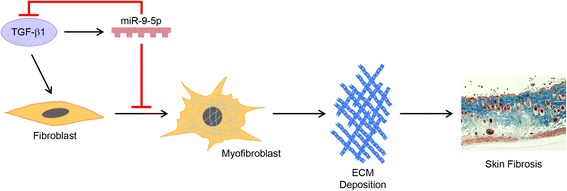
TGF-β1 induces miR-9-5p expression in an attempt to self-limit the promotion of a skin fibrotic program. Over-expression of miR-9-5p in dermal fibroblasts blocks TGFBR2 expression, preventing myofibroblast differentiation and skin fibrosis

Results from the present study suggest that TGF-β1-induced miR-9-5p up-regulation functions as a negative feedback loop in the regulation of TGFBR2 expression in an attempt to reduce the excessive pro-fibrotic signals promoted by TGF-β1 (Fig. 6). This response is however unable to completely counteract fibroblast transformation and skin fibrosis development. Triggering of similarly protective responses seems to underlie the action of other miRNAs like miR-146a, which targets SMAD4 [58]. One reason by which TGF-β1-induced increase in miRNA levels may fail to prevent human dermal fibroblast activation is probably related to the relatively smaller increase of miR-9-5p after TGF-β1 stimulation compared with the magnitude of the response during miR-9-5p over-expression. The level of miR-9-5p was increased 20-fold after treatment with TGF-β1 whereas its levels augmented 40-fold after in vitro transfection (data not shown). It is also possible that biologically relevant up-regulation of miR-9-5p may occur at a later stage than α-SMA expression after TGF-β1 stimulation, thus hampering an effective prevention of this crucial pro-fibrogenic event. Other potential explanations for this limited action include the activation of TGF-β1-independent pro-fibrogenic stimuli, alternative TGF-β1 signaling mediated by receptors other than TGFBR2, and/or signaling through molecules different from Smads. The capacity of miR-9-5p to inhibit the pro-fibrogenic transformation induced by TGF-β1 not only in skin fibrosis but also in pulmonary fibroblasts and peritoneal mesothelial cells [34] confers miR-9-5p a more general counter-regulatory role in organ fibrosis. As TGF-β blockers are not devoid of serious unwanted effects and inhibitory molecules directed towards its inhibition may involve pleiotropic effects, it is tempting to speculate that miR-9-5p could represent an advantageous therapeutic alternative. Nevertheless, off-target effects cannot be excluded, and only large in vivo studies will help to confirm the safety and specificity of miR-9-5p. These particular data in the skin pave the way to explore miR-9-5p as a therapeutic agent. Perhaps in the future, topical miRNA modulators may be of substantive use in dermatology, similarly to the promising P144 (a peptide inhibitor of TGF-β1) whose topical application ameliorates bleomycin-induced skin fibrosis [59].

In addition, miRNAs have been recently reported to circulate in body fluids representing a non-invasive biomarker in the diagnosis, prognosis, and evaluation of skin fibrosis [3]. We found that plasma levels of miR-9-5p in the bleomycin-induced skin fibrosis model were significantly increased. Further correlation between miR-9-5p in skin and/or plasma level and skin fibrosis degree in skin fibrotic connective tissue disorders will determine its potential use as a biomarker. In the skin, the expression of miRNA-7 and miRNA-196a in serum have been shown to inversely correlate with the duration of SSc [28, 60].

In summary, this study supports that miR-9-5p has anti-fibrotic effects in dermal fibroblasts through the down-regulation of the TGF-β pathway via TGFBR2 silencing and provides a novel potential strategy to manage cutaneous fibrotic diseases.

## Conclusions

We report that miR-9-5p expression levels are increased in TGF-β-activated human dermal fibroblasts, while the over-expression of this miRNA abrogates TGF-β signaling through the down-regulation of TGFBR2 expression in these cells. miR-9-5p is also up-regulated in skin and plasma samples from the bleomycin mouse model and shows an anti-fibrotic effect in skin fibrosis, thus providing a novel potential strategy to manage cutaneous fibrotic diseases.

## Methods

### Cell culture and treatments

Human dermal fibroblasts (HDFs) from the American Type Culture Collection (ATCC catalog # PCS-201-012, lot # 58732338) and Invitrogen (Catalog # C-013-5C, lot # 200706-833) were cultured in Dulbecco’s modified Eagle’s medium (DMEM) containing 10 % (volume/volume (vol/vol)) fetal bovine serum (FBS) (HyClone Laboratories, Logan, UT) and supplemented with l-glutamine 2 mM (Life Technologies, CA, USA.) and 1 % (vol/vol) penicillin/streptomycin (Gibco, Rockville, MD). They were kept at 37 °C in the presence of 5 % CO_2_. Treatments with human recombinant TGF-β1 (R&D Systems, Minneapolis, MN) were performed after serum-free starvation of HDFs for 12 h.

### miRNA target prediction bioinformatics tools

Three different and independent online target prediction databases (TargetScan: http://www.targetscan.org/; miRanda: http://www.microrna.org/; and miRwalk: http://zmf.umm.uni-heidelberg.de/apps/zmf/mirwalk/) were used to identify in silico miR-9-5p targets.

### Transfection procedures

HDFs were seeded into 60-mm culture dishes to reach a confluence of 70 %. Where and as indicated, 40-nM pre-miR miRNA precursor of miR-9-5p (pre-miR-9-5p) (AM17100, Ambion, Carlsbad, CA) or 40 nM of a non-targeting sequence pre-miR miRNA precursor negative control #1 (pre-miR-NC) (AM17110, Ambion, Carlsbad, CA), miR inhibitor-9, miR inhibitor-NC (4464088, Ambion Company, USA), and 0.8 % Lipofectamine 2000 (Invitrogen, Carlsbad, CA) were separately mixed in 500 μl of Opti-MEM (Gibco, Grand Island, NY) for 5 minutes (min). Then, the two mixtures were combined and incubated at room temperature for 20 min. The lipofectamine-pre-miRNA mixture was added to the cells and incubated at 37 °C for 6 h. Subsequently, 5 ml fresh medium containing 10 % FBS was added to the culture dishes, and the cells were maintained in culture until used for subsequent experiments.

### Western blotting

Cells were washed in phosphate-buffered saline (PBS) and lysed in 150 μl RIPA lysis buffer containing 150 mM NaCl, 0.1 % sodium dodecyl sulfate (SDS), 1 % sodium deoxycholate, 1 % NP-40, and 25 mM Tris-HCl pH 7.6, in the presence of protease (Complete, Roche Diagnostics, Mannheim, Germany) and phosphatase inhibitors (Sigma-Aldrich, St. Louis, MO). Cells were harvested by scraping, and the samples were clarified by centrifugation at 13,000 rpm for 15 min at 4 °C. A cell fractionation kit (Abcam, Cambridge, USA) was used to isolate cytosolic and nuclear fractions. Protein concentration was measured with the BCA assay (Thermo Scientific, Rockford, IL, USA). Equal amounts of protein (10–50 μg) from the total extract were separated by electrophoresis using an acrylamide/bisacrylamide (10 %) gel and transferred to a nitrocellulose membrane (GE Healthcare, Germany) at 12 V for 20 min in a semi-dry Trans-Blot Turbo system (Bio-Rad, Hercules, CA). After blocking the membranes in 5 % skimmed milk in TBS-T (10 mM), they were incubated with the appropriate primary antibodies, against α-actin (1A4) (1:2000, sc-32251, Santa Cruz Biotechnology, CA, USA), glyceraldehyde-3-phosphate dehydrogenase (GAPDH) (1:20,000; Sigma, St. Louis, MO), fibronectin (1:1000, F7387, Sigma, St. Louis, MO), lamin A/C (1:500, Cell Signaling Technology, Boston, USA), pSmad2, Smad2, and TGFBR2 (1:2000; Santa Cruz Biotechnology, CA, USA) overnight at 4 °C. Membranes were washed in TBS-T (10 mM) and incubated for 1 h at room temperature with secondary fluorescent antibodies IRDye 800 goat anti-rabbit and IRDye 600 goat anti-mouse (1:15,000; LI-COR Biosciences, Lincoln, NE). They were washed with TBS-T (10 mM) and scanned using Odyssey Infrared Imaging system (LI-COR Biosciences, Lincoln, NE). Protein quantity was measured by band intensity using ImageJ 1.48 software.

### Sircol Collagen Assay

Secreted collagen proteins I-V were detected in culture medium using the Sircol Collagen Assay (S1000; Biocolor, Carrickfergus, UK), according to the manufacturer’s instructions.

### Quantification of mRNA and miRNA expression

Total RNA and miRNA were extracted from HDFs or skin samples of mice using miRNeasy Mini Kit (Qiagen, CA, USA) and screened for purity and concentration in a Nanodrop-1000 Spectrophotometer (Thermo Scientific). Plasma miRNA was extracted from blood using miRCURY^TM^ RNA Isolation Kit—Biofluids and UniSp2 Spike-in RNA (Exiqon, Vedbaeck, Denmark). RT was carried out with 500 ng of total RNA using the iScriptTM cDNA Synthesis Kit (Bio-Rad, Hercules, CA). Quantitative RT-PCR was carried out with the iQ™SYBR Green Supermix (Bio-Rad, Hercules, CA), using specific primers for mRNA amplification (Sigma, St. Louis, MO) and miRNA LNA PCR primers for miR-5p (Exiqon, Vedbaeck, Denmark) for miRNA expression analysis. qRT-PCR was performed in a 96-well Bio-Rad CFX96 RT-PCR System with a C1000 Thermal Cycler (Bio-Rad, Hercules, CA). A Ct value was obtained from each amplification curve using CFX96 Analysis Software provided by the manufacturer. Relative mRNA and miRNA expression was determined using the 2^−ΔΔCt^ method [61]. GAPDH, snRNAU6, or UniSp-2 gene expression levels in each sample were used for normalization, respectively. The sequences of the primers were human GAPDH: forward: 5′-GAGTCAACGGATTTGGTCGT-3′, reverse: 5′-TTGATTTTGGAGGGATCTCG-3′; human Smad7: forward: 5′-AGAGGCTGTGTTGCTGTGAA-3′, reverse: 5′-AAATCCATCGGGTATCTGGA-3′; human TGFBR1: forward: 5′-TGGAGAGGAAAGTGGCGGGGAG-3′, reverse: 5′-GCCTCACGGAACCACGAACG-3′; human Col1α1: forward: 5′-CGGACGACCTGGTGAGAGA-3′, reverse: 5′-CATTGTGTCCCCTAATGCCTT-3′; human FN: forward: 5′-GTGTTGGGAATGGTCGTGGGGAATG-3′, reverse: 5′-CCAATGCCACGGCCATAGCAGTAGC-3′; human α-SMA: forward: 5′-TTCAATGTCCCAGCCATGTA-3′, reverse: 5′-GAAGGAATAGCCACGCTCAG-3′; and human TGFBR2: forward: 5′-CCATGTCTCACAGCCAGCTA-3′, reverse: 5′-CCAGGAGAAATAAGGGCACA-3′.

### Immunofluorescence cell staining

HDF cells were seeded onto 24-well plates containing 10-mm-diameter glass coverslips. They were transfected with 40-nM pre-miRs. After 24-h starvation, cells were incubated with 5 ng/ml TGF-β1 (R&D Systems, Minneapolis, MN) for the indicated times. After treatments, cells were washed briefly with cold PBS for three times and fixed with 4 % paraformaldehyde for 20 min. Then, the cell membrane was permeabilized by Triton X-100 (0.25 %) in PBS for 5 min and blocked by FBS (10 %) for 1 h, at 37 °C. The cells were incubated with anti-α-actin (1A4) (1:2000, sc-32251, Santa Cruz Biotechnology) or anti-Smad2/3 (1:1000, 610843, BD Biosciences) overnight at 4 °C and subsequently incubated with FITC-conjugated anti-mouse or goat anti-rabbit antibody for 1 h. Cells were washed with PBS and nuclei were stained with 4′,6-diamidino-2-phenylindole (DAPI) and F-actin with phalloidin (1:1000, Roche Molecular Biochemicals, Mannheim, Germany) for 5 min at room temperature. The coverslips were mounted on slides using Mowiol (Calbiochem, Nottingham, U.K.). Immunofluorescence was analyzed using a fluorescence microscope (Nikon 80i, Japan).

### Lentiviral vector construct

The lentivector-based miRNA precursor constructs expressing the miR-9-5p (lenti-miR-9) (MMIR-9-1-PA-1) and the scramble negative control (lenti-SC) (MMIR-000-PA-1) were purchased from SBI System Biosciences (Mountain View, CA). Pseudoviral particles were prepared using the pPACK-F1 Lentivector packaging system (SBI System Biosciences, Mountain View, CA) and HEK 293T producer cell line. The titration of pseudoviral particles generated with the lentiviral vectors was determined by calculating the percentage of positive green fluorescent protein (GFP) expression cells by flow cytometry in a BD FACS CantoTM II High Throughput Sampler flow cytometer (Becton Dickinson Bioscience, Franklin Lakes, NJ) 72 h after infection. The lentivirus titers were calculated as described [62] and expressed in infection units (ifu)/ml.

### Mouse model of bleomycin-induced dermal fibrosis and in vivo delivery of lentiviral vectors

Mice were housed in the animal facility at CBMSO, in accordance with EU regulations. Skin fibrosis was induced in C57BL/6 mice by daily subcutaneous injections of bleomycin, in accordance with previously published protocols [63, 64]. A dose of 1 × 10^8^ ifu/ml of lentivirus (lenti-miR-9 or lenti-SC) in 40 μl saline serum was subcutaneously delivered 4 days before bleomycin administration. Two groups of six mice were injected on a daily basis with 10 μg bleomycin (Sigma, St. Louis, MO) for 4 weeks, respectively. Saline solution was used as vehicle in control groups of mice. Mice were killed by cervical dislocation, and the back skin was removed and processed for histological examination. Animals were handled in agreement with the Guide for the Care and Use of Laboratory Animals contained in Directive 2010/63/EU of the European Parliament. Approval was granted by the local ethics review board of the Centro de Biología Molecular “Severo Ochoa.”

### Hematoxylin and eosin and Masson’s trichrome stainings

Skin samples of mice in the bleomycin or saline group were quickly dissected and immersed in 4 % neutral buffered formalin for 24 h and stained with Masson’s trichrome or hematoxylin/eosin staining as described previously [65].

### Statistical analysis

Data were analyzed using non-parametric tests. The difference between two independent groups was examined with Mann-Whitney test, while more than two groups were compared with Kruskall-Wallis test. A *P* value of 0.05 or less was considered statistically significant. Data were analyzed using GraphPad Prism 5.0 (GraphPad Software, La Jolla, CA, USA).

## Additional file

Additional file 1: Figure S1. miR-9-5p does not affect TGF-β-induced TGFBR1 and Smad7 expression and attenuates long-term Smad2 phosphorylation. (A) qRT-PCR analysis for mRNA expression of TGFBR1 in HDF transfected with 40-nM pre-miR-9-5p or pre-miR-NC treated with TGF-β1 (5 ng/ml) for the indicated times. (B) qRT-PCR analysis for mRNA expression of Smad7 in HDF transfected with 40-nM pre-miR-9-5p or pre-miR-NC treated with TGF-β1 (5 ng/ml) for the indicated times. The bar graph shows values after correction by GAPDH expression and normalized to control conditions. (C) Western blot analysis (left) and quantification (right) of pSmad2 protein levels in HDF cells transfected with 40-nM pre-miR-9-5p or pre-miR-NC and treated with TGF-β1 (5 ng/ml) at the indicated times (a.u., arbitrary units). The bar graph shows values after correction by Smad2 expression and normalized to control conditions. All bar graphs represent mean ± SEM of three independent experiments, **P* < 0.001 and ****P* < 0.01 compared to control cells and ^#^ *P* < 0.05 compared to its corresponding negative control time point.

## Abbreviations

3′-UTR: 3′-untraslated region
ATCC: American Type Culture Collection
Col1α1: collagen, type I, alpha 1
co-Smad: common-mediator Smad
DAPI: 4′,6-diamidino-2-phenylindole
DMEM: Dulbecco’s modified Eagle’s medium
ECM: extracellular matrix
EMT: epithelial to mesenchymal transition
EndoMT: endothelial to mesenchymal transition
FBS: fetal bovine serum
FN: fibronectin
GAPDH: glyceraldehyde-3-phosphate dehydrogenase
GFP: green fluorescent protein
h: hours
H&E: hematoxylin and eosin
HDFs: human dermal fibroblasts
ifu/ml: infection units/milliliter
lenti-miR-9: lentivector expressing miR-9-5p
lenti-SC: lentivector expressing the scramble negative control
LOXs: lysyl oxidases
min: minutes
miR-9-5p: microRNA-9-5p
miRNAs: microRNAs
MMPs: matrix metalloproteases
mRNA: messenger RNA
NOX4: NADPH oxidase 4
PBS: phosphate-buffered saline
pre-miR-9-5p: miR-9-5p precursor
pre-miR-NC: negative control precursor
pri-miRNA: primary miRNA transcript
qRT-PCR: quantitative reverse transcription-polymerase chain reaction
ROS: reactive oxygen species
R-Smad: receptor-specific Smad
SBEs: Smad-binding elements
SDS: sodium dodecyl sulfate
SSc: systemic sclerosis
TGFBI: transforming growth factor, beta-induced
TGFBR1: type I TGF-β receptor
TGFBR2: type II TGF-β receptor
TGF-β: transforming growth factor-β
vol/vol: volume/volume
α-SMA: alpha-smooth muscle actin
