# Supplementary figures and images for: Protective role for miR-9-5p in the fibrogenic transformation of human dermal fibroblasts
Source: Fibrogenesis Tissue Repair. 2016 May 10;9:7. doi: 10.1186/s13069-016-0044-2 (PMC4891847; doi:10.1186/s13069-016-0044-2)

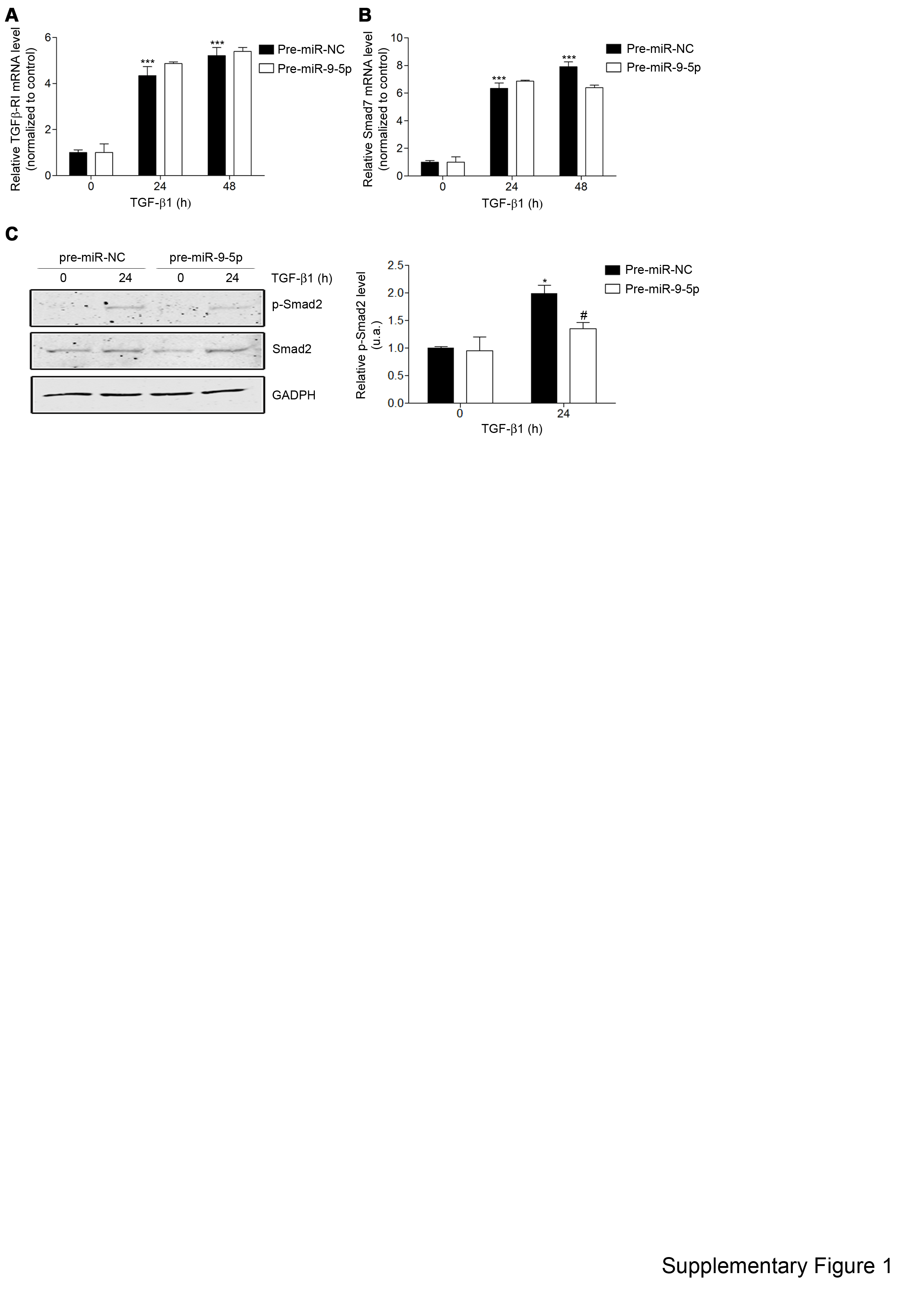

Supplement: Additional file 1: Figure S1. — miR-9-5p does not affect TGF-β-induced TGFBR1 and Smad7 expression and attenuates long-term Smad2 phosphorylation. (A) qRT-PCR analysis for mRNA expression of TGFBR1 in HDF transfected with 40-nM pre-miR-9-5p or pre-miR-NC treated with TGF-β1 (5 ng/ml) for the indicated times. (B) qRT-PCR analysis for mRNA expression of Smad7 in HDF transfected with 40-nM pre-miR-9-5p or pre-miR-NC treated with TGF-β1 (5 ng/ml) for the indicated times. The bar graph shows values after correction by GAPDH expression and normalized to control conditions. (C) Western blot analysis (left) and quantification (right) of pSmad2 protein levels in HDF cells transfected with 40-nM pre-miR-9-5p or pre-miR-NC and treated with TGF-β1 (5 ng/ml) at the indicated times (a.u., arbitrary units). The bar graph shows values after correction by Smad2 expression and normalized to control conditions. All bar graphs represent mean ± SEM of three independent experiments, *P < 0.001 and ***P < 0.01 compared to control cells and # P < 0.05 compared to its corresponding negative control time point. [file 13069_2016_44_MOESM1_ESM.tif]
